# (6-Chloropyridazin-3-yl)ferrocene

**DOI:** 10.1107/S1600536813031310

**Published:** 2013-12-04

**Authors:** Guo-Qing Shi, Wen-En Zhao, Xiao-Hui Zhao, Lu-Ye Hao

**Affiliations:** a School of Chemical Engineering and Energy, Zhengzhou University, Henan, Zhengzhou, 450052, People’s Republic of China; b School of Food and Bioengineering, Zhengzhou University of Light Industry, Henan, Zhengzhou, 450052, People’s Republic of China; c Henan Industrial University Chemical Technology Vocational College, Henan, Zhengzhou, 450052, People’s Republic of China

## Abstract

The asymmetric unit of the title compound, [Fe(C_5_H_5_)(C_9_H_6_ClN_2_)], contains two independent mol­ecules in which the cyclo­penta­dienyl rings are almost parallel, making dihedral angles of 2.16 (4) and 2.71 (5), and the dihedral angles between the pyridazinyl and substituted cyclo­penta­dienyl rings are 9.65 (5) and 11.53 (8)°. In the crystal, mol­ecules are linked by C—H⋯N hydrogen bonds into chains along the *c-*axis direction.

## Related literature   

For the synthesis of the title compound, see: Xu *et al.* (2012 ▶). For applications of organomercury compounds, see: Beletskaya *et al.* (2001 ▶); Tsvetkov *et al.* (2000 ▶); Xu *et al.* (2010 ▶); For palladium-catalysed reactions, see: Meijere & Diederich (2004 ▶).
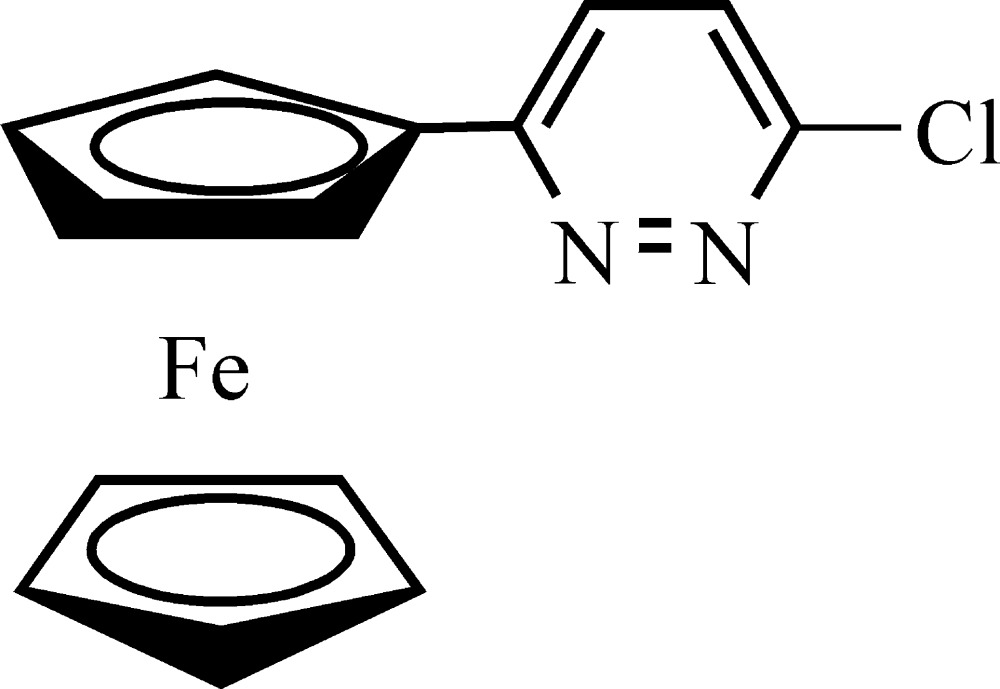


## Experimental   

### 

#### Crystal data  

[Fe(C_5_H_5_)(C_9_H_6_ClN_2_)]
*M* *_r_* = 298.55
Monoclinic, 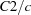

*a* = 20.5488 (19) Å
*b* = 12.3788 (6) Å
*c* = 23.043 (2) Å
β = 122.843 (13)°
*V* = 4924.5 (7) Å^3^
*Z* = 16
Mo *K*α radiation
μ = 1.42 mm^−1^
*T* = 291 K
0.30 × 0.30 × 0.25 mm

#### Data collection  

Oxford Diffraction Xcalibur Eos Gemini diffractometer
Absorption correction: multi-scan (*CrysAlis PRO*; Agilent, 2011 ▶) *T*_min_ = 0.675, *T*_max_ = 0.718
18413 measured reflections
4575 independent reflections
3783 reflections with *I* > 2σ(*I*)
*R*_int_ = 0.032

#### Refinement  

*R*[*F* ^2^ > 2σ(*F* ^2^)] = 0.031
*wR*(*F* ^2^) = 0.078
*S* = 1.04
4575 reflections
325 parameters
H-atom parameters constrained
Δρ_max_ = 0.36 e Å^−3^
Δρ_min_ = −0.23 e Å^−3^

### 

Data collection: *CrysAlis PRO* (Agilent, 2011 ▶); cell refinement: *CrysAlis PRO*; data reduction: *CrysAlis PRO*; program(s) used to solve structure: *SHELXS97* (Sheldrick, 2008 ▶); program(s) used to refine structure: *SHELXL97* (Sheldrick, 2008 ▶); molecular graphics: *SHELXTL* (Sheldrick, 2008 ▶); software used to prepare material for publication: *SHELXTL*.

## Supplementary Material

Crystal structure: contains datablock(s) global, I. DOI: 10.1107/S1600536813031310/rn2116sup1.cif

Structure factors: contains datablock(s) I. DOI: 10.1107/S1600536813031310/rn2116Isup2.hkl

Additional supporting information:  crystallographic information; 3D view; checkCIF report

## Figures and Tables

**Table 1:** Hydrogen-bond geometry (Å, °)

*D*—H⋯*A*	*D*—H	H⋯*A*	*D*⋯*A*	*D*—H⋯*A*
C2—H2⋯N3^i^	0.98	2.55	3.454 (4)	153
C12—H12⋯N4^i^	0.93	2.41	3.320 (4)	166
C26—H26⋯N2^ii^	0.93	2.43	3.302 (4)	156

Symmetry codes: (i) 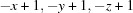; (ii) 
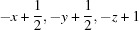
.

**Fig. 1.**
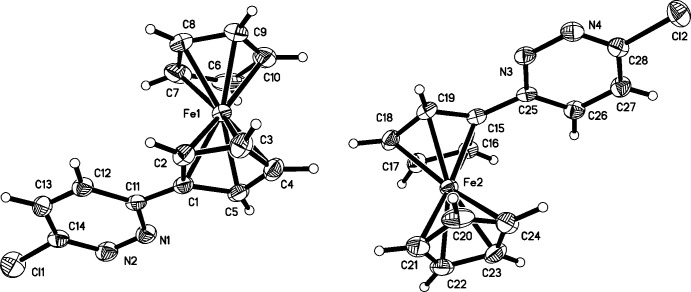
The molecular structure of the title compound with displacement ellipsoids at the 30% probability level.

**Fig. 2.**
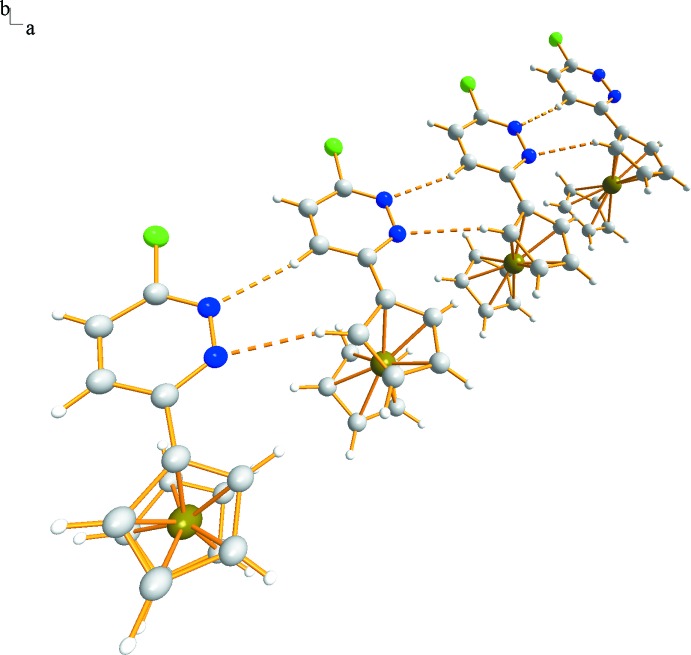
The crystal packing of the molecules viewed down c axis.

## supplementary crystallographic information

### 1. Comment

Palladium catalyzed coupling reactions are widely used and powerful tools in organic synthesis (Meijere & Diederich, 2004). The organomercury compounds have a number of notable advantages over other organometallic compounds commonly used in cross-coupling reactions, including higher selectivity of reactions, extra stability, as well as easy availability by a direct mercuration of ferrocene (Beletskaya *et al.*, 2001; Tsvetkov *et al.*, 2000; Xu *et al.*, 2010). The coupling reaction of with chloromercuriferrocene and 3,6-dichloropyridazine readily afforded the title compound.

There are two molecules in the asymmetric unit(Fig.1). The two cyclopentadienyl rings are almost parallel with dihedral angles of 2.16 (4)° and 2.71 (5)° for molecules containing Fe2 or Fe1, respectively. The dihedral angle between the pyridazinyl and substituted cyclopentadienyl ring is 9.65 (5)° and 11.53 (8)° for molecules containing Fe1 or Fe2, respectively. Intermolecular C—H···N hydrogen bonds construct a chain along the *c* axis direction (Fig.2).

### 2. Experimental

The title compound was obtained from the coupling reaction of chloromercuriferrocene and 3,6-dichloropyridazine as described in literature (Xu *et al.*, 2012) and recrystallized from dichloromethane-petroleum ether solution at room temperature to give the desired crystals suitable for single-crystal X-ray diffraction.

### 3. Refinement

H atoms attached to C atoms of the title compound were placed in geometrically idealized positions and treated as riding with C—H distances constrained to 0.93–0.96 Å.

### Crystal data

**Table:**

[Fe(C_5_H_5_)(C_9_H_6_ClN_2_)]	*F*(000) = 2432
*M**_r_* = 298.55	*D*_x_ = 1.611 Mg m^−^^3^
Monoclinic, *C*2/*c*	Mo *K*α radiation, λ = 0.71073 Å
*a* = 20.5488 (19) Å	Cell parameters from 5464 reflections
*b* = 12.3788 (6) Å	θ = 3.1–26.3°
*c* = 23.043 (2) Å	µ = 1.42 mm^−^^1^
β = 122.843 (13)°	*T* = 291 K
*V* = 4924.5 (7) Å^3^	Block, brown
*Z* = 16	0.30 × 0.30 × 0.25 mm

### Data collection

**Table:**

Oxford Diffraction Xcalibur Eos Gemini diffractometer	4575 independent reflections
Radiation source: Enhance (Mo) X-ray Source	3783 reflections with *I* > 2σ(*I*)
Graphite monochromator	*R*_int_ = 0.032
Detector resolution: 16.2312 pixels mm^-1^	θ_max_ = 25.5°, θ_min_ = 3.2°
ω scans	*h* = −23→24
Absorption correction: multi-scan (*CrysAlis PRO*; Agilent, 2011)	*k* = −14→14
*T*_min_ = 0.675, *T*_max_ = 0.718	*l* = −27→27
18413 measured reflections	

### Refinement

**Table:**

Refinement on *F*^2^	Primary atom site location: structure-invariant direct methods
Least-squares matrix: full	Secondary atom site location: difference Fourier map
*R*[*F*^2^ > 2σ(*F*^2^)] = 0.031	Hydrogen site location: inferred from neighbouring sites
*wR*(*F*^2^) = 0.078	H-atom parameters constrained
*S* = 1.04	*w* = 1/[σ^2^(*F*_o_^2^) + (0.0332*P*)^2^ + 3.3249*P*] where *P* = (*F*_o_^2^ + 2*F*_c_^2^)/3
4575 reflections	(Δ/σ)_max_ = 0.001
325 parameters	Δρ_max_ = 0.36 e Å^−^^3^
0 restraints	Δρ_min_ = −0.23 e Å^−^^3^

### Special details

**Table:**

Geometry. All e.s.d.'s (except the e.s.d. in the dihedral angle between two l.s. planes)are estimated using the full covariance matrix. The cell e.s.d.'s are takeninto account individually in the estimation of e.s.d.'s in distances, anglesand torsion angles; correlations between e.s.d.'s in cell parameters are onlyused when they are defined by crystal symmetry. An approximate (isotropic)treatment of cell e.s.d.'s is used for estimating e.s.d.'s involving l.s. planes.
Refinement. Refinement of *F*^2^ against ALL reflections. The weighted *R*-factor *wR* andgoodness of fit *S* are based on *F*^2^, conventional *R*-factors *R* are basedon *F*, with *F* set to zero for negative *F*^2^. The threshold expression of*F*^2^ > σ(*F*^2^) is used only for calculating *R*-factors(gt) *etc*. and isnot relevant to the choice of reflections for refinement. *R*-factors basedon *F*^2^ are statistically about twice as large as those based on *F*, and *R*-factors based on ALL data will be even larger.

### Fractional atomic coordinates and isotropic or equivalent isotropic displacement parameters (Å^2^)

**Table:**

	*x*	*y*	*z*	*U*_iso_*/*U*_eq_	
Fe1	0.542789 (17)	0.30107 (3)	0.582945 (16)	0.03750 (11)	
Fe2	0.203696 (17)	0.43834 (2)	0.410109 (15)	0.03501 (10)	
Cl1	0.65041 (5)	−0.24694 (6)	0.71490 (4)	0.0727 (2)	
Cl2	0.11431 (5)	0.99199 (6)	0.30309 (4)	0.0690 (2)	
N1	0.52234 (11)	−0.00916 (17)	0.60350 (11)	0.0485 (5)	
N2	0.54980 (12)	−0.09968 (17)	0.64197 (12)	0.0514 (5)	
N3	0.23719 (10)	0.74910 (16)	0.41199 (10)	0.0422 (5)	
N4	0.21252 (11)	0.84273 (16)	0.37651 (11)	0.0458 (5)	
C1	0.53402 (12)	0.14578 (19)	0.54950 (12)	0.0404 (5)	
C2	0.56826 (14)	0.2140 (2)	0.52259 (12)	0.0458 (6)	
H2	0.6197	0.2050	0.5301	0.055*	
C3	0.51425 (15)	0.2960 (2)	0.48304 (13)	0.0528 (7)	
H3	0.5222	0.3545	0.4589	0.063*	
C4	0.44721 (14)	0.2807 (2)	0.48506 (13)	0.0528 (7)	
H4	0.4010	0.3267	0.4626	0.063*	
C5	0.45863 (13)	0.1893 (2)	0.52575 (13)	0.0456 (6)	
H5	0.4217	0.1603	0.5362	0.055*	
C6	0.5420 (2)	0.3381 (3)	0.66884 (17)	0.0745 (10)	
H6	0.5080	0.3067	0.6820	0.089*	
C7	0.6158 (2)	0.2982 (2)	0.68728 (14)	0.0697 (9)	
H7	0.6417	0.2342	0.7155	0.084*	
C8	0.64510 (15)	0.3671 (2)	0.65830 (14)	0.0572 (7)	
H8	0.6951	0.3597	0.6627	0.069*	
C9	0.59049 (16)	0.4488 (2)	0.62266 (15)	0.0577 (7)	
H9	0.5955	0.5079	0.5971	0.069*	
C10	0.52710 (17)	0.4316 (2)	0.62874 (16)	0.0647 (8)	
H10	0.4805	0.4765	0.6084	0.078*	
C11	0.56917 (12)	0.05099 (18)	0.59310 (12)	0.0383 (5)	
C12	0.64703 (13)	0.0226 (2)	0.62229 (13)	0.0440 (6)	
H12	0.6793	0.0657	0.6153	0.053*	
C13	0.67469 (14)	−0.0690 (2)	0.66087 (13)	0.0472 (6)	
H13	0.7258	−0.0911	0.6810	0.057*	
C14	0.62236 (14)	−0.12723 (19)	0.66835 (12)	0.0442 (6)	
C15	0.21916 (12)	0.58613 (18)	0.45514 (11)	0.0361 (5)	
C16	0.18188 (13)	0.51278 (19)	0.47649 (11)	0.0399 (5)	
H16	0.1308	0.5228	0.4695	0.048*	
C17	0.23183 (13)	0.4242 (2)	0.50975 (12)	0.0444 (6)	
H17	0.2209	0.3615	0.5292	0.053*	
C18	0.29996 (13)	0.4399 (2)	0.50910 (12)	0.0453 (6)	
H18	0.3440	0.3902	0.5279	0.054*	
C19	0.29259 (12)	0.53913 (19)	0.47570 (11)	0.0412 (5)	
H19	0.3308	0.5702	0.4675	0.049*	
C20	0.20433 (19)	0.4178 (3)	0.32253 (14)	0.0656 (8)	
H20	0.2404	0.4514	0.3126	0.079*	
C21	0.21428 (17)	0.3175 (2)	0.35522 (14)	0.0594 (7)	
H21	0.2587	0.2690	0.3723	0.071*	
C22	0.14951 (16)	0.2994 (2)	0.35885 (13)	0.0562 (7)	
H22	0.1410	0.2358	0.3793	0.067*	
C23	0.09880 (15)	0.3871 (2)	0.32879 (13)	0.0564 (7)	
H23	0.0487	0.3953	0.3241	0.068*	
C24	0.13262 (18)	0.4609 (2)	0.30618 (13)	0.0621 (8)	
H24	0.1102	0.5299	0.2829	0.074*	
C25	0.18747 (12)	0.68643 (17)	0.41683 (11)	0.0343 (5)	
C26	0.10951 (13)	0.71658 (19)	0.38661 (12)	0.0427 (6)	
H26	0.0754	0.6723	0.3905	0.051*	
C27	0.08504 (14)	0.8115 (2)	0.35173 (13)	0.0470 (6)	
H27	0.0342	0.8353	0.3312	0.056*	
C28	0.14001 (14)	0.87131 (19)	0.34830 (12)	0.0424 (6)	

### Atomic displacement parameters (Å^2^)

**Table:**

	*U*^11^	*U*^22^	*U*^33^	*U*^12^	*U*^13^	*U*^23^
Fe1	0.03557 (18)	0.03842 (19)	0.03580 (19)	−0.00429 (14)	0.01758 (15)	−0.00388 (14)
Fe2	0.03542 (18)	0.03567 (19)	0.03032 (18)	−0.00343 (13)	0.01547 (14)	−0.00137 (14)
Cl1	0.0749 (5)	0.0669 (5)	0.0773 (5)	−0.0009 (4)	0.0419 (4)	0.0199 (4)
Cl2	0.0785 (5)	0.0568 (4)	0.0694 (5)	0.0043 (4)	0.0386 (4)	0.0203 (4)
N1	0.0378 (11)	0.0504 (13)	0.0625 (14)	−0.0083 (9)	0.0306 (10)	−0.0079 (11)
N2	0.0472 (12)	0.0534 (13)	0.0617 (14)	−0.0125 (10)	0.0348 (11)	−0.0065 (11)
N3	0.0360 (10)	0.0450 (12)	0.0488 (12)	−0.0090 (9)	0.0251 (9)	−0.0034 (10)
N4	0.0474 (12)	0.0457 (12)	0.0513 (13)	−0.0103 (10)	0.0312 (10)	−0.0015 (10)
C1	0.0343 (12)	0.0448 (13)	0.0409 (13)	−0.0060 (10)	0.0195 (10)	−0.0123 (11)
C2	0.0416 (13)	0.0578 (16)	0.0394 (13)	−0.0023 (12)	0.0230 (11)	−0.0063 (12)
C3	0.0499 (15)	0.0681 (18)	0.0377 (14)	0.0025 (13)	0.0219 (12)	0.0024 (13)
C4	0.0393 (13)	0.0653 (17)	0.0389 (14)	0.0044 (12)	0.0116 (11)	−0.0041 (13)
C5	0.0331 (12)	0.0510 (15)	0.0468 (14)	−0.0087 (11)	0.0179 (11)	−0.0134 (12)
C6	0.101 (3)	0.080 (2)	0.067 (2)	−0.046 (2)	0.061 (2)	−0.0397 (18)
C7	0.099 (2)	0.0494 (17)	0.0309 (14)	−0.0075 (16)	0.0161 (15)	−0.0060 (13)
C8	0.0437 (14)	0.0570 (17)	0.0541 (17)	−0.0112 (13)	0.0154 (13)	−0.0136 (14)
C9	0.0603 (17)	0.0383 (14)	0.0687 (19)	−0.0101 (13)	0.0312 (15)	−0.0052 (13)
C10	0.0603 (18)	0.0564 (18)	0.075 (2)	−0.0028 (14)	0.0349 (16)	−0.0246 (16)
C11	0.0355 (12)	0.0412 (13)	0.0429 (13)	−0.0113 (10)	0.0242 (10)	−0.0158 (11)
C12	0.0381 (12)	0.0475 (14)	0.0550 (15)	−0.0094 (11)	0.0308 (12)	−0.0069 (12)
C13	0.0402 (13)	0.0500 (15)	0.0568 (16)	−0.0018 (11)	0.0298 (12)	−0.0032 (12)
C14	0.0488 (14)	0.0449 (14)	0.0423 (14)	−0.0083 (11)	0.0269 (12)	−0.0082 (11)
C15	0.0351 (12)	0.0399 (12)	0.0342 (12)	−0.0085 (10)	0.0194 (10)	−0.0068 (10)
C16	0.0400 (12)	0.0450 (13)	0.0384 (13)	−0.0051 (10)	0.0237 (11)	−0.0044 (11)
C17	0.0470 (14)	0.0505 (15)	0.0320 (12)	−0.0020 (11)	0.0189 (11)	0.0037 (11)
C18	0.0376 (12)	0.0543 (15)	0.0327 (12)	0.0034 (11)	0.0117 (10)	0.0004 (11)
C19	0.0327 (12)	0.0509 (14)	0.0370 (13)	−0.0087 (10)	0.0169 (10)	−0.0086 (11)
C20	0.084 (2)	0.077 (2)	0.0485 (16)	−0.0356 (18)	0.0438 (16)	−0.0257 (15)
C21	0.0629 (18)	0.0566 (17)	0.0503 (16)	0.0016 (14)	0.0252 (14)	−0.0178 (14)
C22	0.0673 (18)	0.0392 (14)	0.0451 (15)	−0.0158 (13)	0.0194 (14)	−0.0058 (12)
C23	0.0431 (14)	0.0668 (18)	0.0394 (14)	−0.0093 (13)	0.0094 (11)	−0.0068 (13)
C24	0.086 (2)	0.0470 (15)	0.0294 (13)	0.0004 (15)	0.0158 (14)	0.0025 (12)
C25	0.0355 (11)	0.0377 (12)	0.0346 (12)	−0.0112 (9)	0.0221 (10)	−0.0112 (10)
C26	0.0373 (12)	0.0453 (14)	0.0527 (15)	−0.0080 (11)	0.0291 (11)	−0.0032 (12)
C27	0.0376 (13)	0.0530 (15)	0.0528 (15)	0.0003 (11)	0.0260 (12)	0.0006 (12)
C28	0.0508 (14)	0.0411 (13)	0.0380 (13)	−0.0037 (11)	0.0259 (11)	−0.0019 (11)

### Geometric parameters (Å, º)

**Table:**

Fe1—C7	2.030 (3)	C6—C7	1.423 (4)
Fe1—C8	2.036 (3)	C6—H6	0.9800
Fe1—C2	2.039 (2)	C7—C8	1.405 (4)
Fe1—C6	2.041 (3)	C7—H7	0.9800
Fe1—C9	2.042 (3)	C8—C9	1.398 (4)
Fe1—C5	2.042 (2)	C8—H8	0.9800
Fe1—C1	2.042 (2)	C9—C10	1.400 (4)
Fe1—C3	2.045 (3)	C9—H9	0.9800
Fe1—C10	2.049 (3)	C10—H10	0.9800
Fe1—C4	2.050 (2)	C11—C12	1.403 (3)
Fe2—C16	2.033 (2)	C12—C13	1.360 (3)
Fe2—C24	2.038 (3)	C12—H12	0.9300
Fe2—C20	2.041 (3)	C13—C14	1.381 (3)
Fe2—C22	2.041 (2)	C13—H13	0.9300
Fe2—C15	2.041 (2)	C15—C16	1.436 (3)
Fe2—C23	2.044 (2)	C15—C19	1.437 (3)
Fe2—C21	2.045 (3)	C15—C25	1.456 (3)
Fe2—C19	2.045 (2)	C16—C17	1.411 (3)
Fe2—C17	2.049 (2)	C16—H16	0.9800
Fe2—C18	2.055 (2)	C17—C18	1.422 (3)
Cl1—C14	1.734 (3)	C17—H17	0.9800
Cl2—C28	1.732 (2)	C18—C19	1.413 (3)
N1—C11	1.337 (3)	C18—H18	0.9800
N1—N2	1.348 (3)	C19—H19	0.9800
N2—C14	1.314 (3)	C20—C21	1.408 (4)
N3—C25	1.335 (3)	C20—C24	1.413 (4)
N3—N4	1.349 (3)	C20—H20	0.9800
N4—C28	1.310 (3)	C21—C22	1.396 (4)
C1—C2	1.437 (3)	C21—H21	0.9800
C1—C5	1.438 (3)	C22—C23	1.400 (4)
C1—C11	1.456 (3)	C22—H22	0.9800
C2—C3	1.413 (4)	C23—C24	1.408 (4)
C2—H2	0.9800	C23—H23	0.9800
C3—C4	1.416 (4)	C24—H24	0.9800
C3—H3	0.9800	C25—C26	1.407 (3)
C4—C5	1.405 (4)	C26—C27	1.357 (3)
C4—H4	0.9800	C26—H26	0.9300
C5—H5	0.9800	C27—C28	1.388 (3)
C6—C10	1.407 (4)	C27—H27	0.9300
			
C7—Fe1—C8	40.43 (12)	C4—C5—Fe1	70.22 (14)
C7—Fe1—C2	120.04 (12)	C1—C5—Fe1	69.38 (12)
C8—Fe1—C2	105.41 (11)	C4—C5—H5	125.9
C7—Fe1—C6	40.93 (13)	C1—C5—H5	125.9
C8—Fe1—C6	68.32 (12)	Fe1—C5—H5	125.9
C2—Fe1—C6	156.98 (14)	C10—C6—C7	107.3 (3)
C7—Fe1—C9	67.54 (12)	C10—C6—Fe1	70.17 (17)
C8—Fe1—C9	40.12 (11)	C7—C6—Fe1	69.10 (16)
C2—Fe1—C9	122.72 (11)	C10—C6—H6	126.3
C6—Fe1—C9	67.57 (12)	C7—C6—H6	126.3
C7—Fe1—C5	125.15 (12)	Fe1—C6—H6	126.3
C8—Fe1—C5	160.73 (11)	C8—C7—C6	108.1 (3)
C2—Fe1—C5	69.05 (10)	C8—C7—Fe1	70.01 (15)
C6—Fe1—C5	109.19 (11)	C6—C7—Fe1	69.96 (17)
C9—Fe1—C5	158.25 (11)	C8—C7—H7	126.0
C7—Fe1—C1	106.73 (11)	C6—C7—H7	126.0
C8—Fe1—C1	122.61 (10)	Fe1—C7—H7	126.0
C2—Fe1—C1	41.24 (9)	C9—C8—C7	107.7 (3)
C6—Fe1—C1	122.15 (12)	C9—C8—Fe1	70.17 (15)
C9—Fe1—C1	159.18 (10)	C7—C8—Fe1	69.55 (15)
C5—Fe1—C1	41.24 (9)	C9—C8—H8	126.2
C7—Fe1—C3	155.42 (13)	C7—C8—H8	126.2
C8—Fe1—C3	120.36 (12)	Fe1—C8—H8	126.2
C2—Fe1—C3	40.47 (10)	C8—C9—C10	109.0 (3)
C6—Fe1—C3	161.87 (14)	C8—C9—Fe1	69.71 (15)
C9—Fe1—C3	107.86 (12)	C10—C9—Fe1	70.26 (15)
C5—Fe1—C3	68.12 (11)	C8—C9—H9	125.5
C1—Fe1—C3	68.52 (10)	C10—C9—H9	125.5
C7—Fe1—C10	67.98 (13)	Fe1—C9—H9	125.5
C8—Fe1—C10	67.80 (11)	C9—C10—C6	108.0 (3)
C2—Fe1—C10	159.77 (12)	C9—C10—Fe1	69.72 (15)
C6—Fe1—C10	40.25 (13)	C6—C10—Fe1	69.58 (16)
C9—Fe1—C10	40.03 (11)	C9—C10—H10	126.0
C5—Fe1—C10	123.52 (11)	C6—C10—H10	126.0
C1—Fe1—C10	158.49 (11)	Fe1—C10—H10	126.0
C3—Fe1—C10	125.08 (12)	N1—C11—C12	121.3 (2)
C7—Fe1—C4	162.27 (13)	N1—C11—C1	115.9 (2)
C8—Fe1—C4	156.62 (12)	C12—C11—C1	122.9 (2)
C2—Fe1—C4	68.39 (10)	C13—C12—C11	119.1 (2)
C6—Fe1—C4	125.99 (13)	C13—C12—H12	120.5
C9—Fe1—C4	122.96 (11)	C11—C12—H12	120.5
C5—Fe1—C4	40.17 (10)	C12—C13—C14	116.0 (2)
C1—Fe1—C4	68.47 (10)	C12—C13—H13	122.0
C3—Fe1—C4	40.46 (10)	C14—C13—H13	122.0
C10—Fe1—C4	109.84 (12)	N2—C14—C13	124.9 (2)
C16—Fe2—C24	120.79 (11)	N2—C14—Cl1	114.90 (18)
C16—Fe2—C20	157.30 (12)	C13—C14—Cl1	120.2 (2)
C24—Fe2—C20	40.53 (12)	C16—C15—C19	106.9 (2)
C16—Fe2—C22	122.93 (11)	C16—C15—C25	126.9 (2)
C24—Fe2—C22	67.50 (11)	C19—C15—C25	126.12 (19)
C20—Fe2—C22	67.47 (11)	C16—C15—Fe2	69.06 (12)
C16—Fe2—C15	41.28 (9)	C19—C15—Fe2	69.57 (13)
C24—Fe2—C15	106.80 (10)	C25—C15—Fe2	124.11 (15)
C20—Fe2—C15	122.10 (11)	C17—C16—C15	108.2 (2)
C22—Fe2—C15	159.23 (10)	C17—C16—Fe2	70.37 (13)
C16—Fe2—C23	106.09 (10)	C15—C16—Fe2	69.67 (12)
C24—Fe2—C23	40.36 (11)	C17—C16—H16	125.9
C20—Fe2—C23	67.91 (12)	C15—C16—H16	125.9
C22—Fe2—C23	40.09 (11)	Fe2—C16—H16	125.9
C15—Fe2—C23	122.70 (10)	C16—C17—C18	108.5 (2)
C16—Fe2—C21	159.65 (11)	C16—C17—Fe2	69.19 (13)
C24—Fe2—C21	67.82 (12)	C18—C17—Fe2	69.96 (13)
C20—Fe2—C21	40.32 (12)	C16—C17—H17	125.7
C22—Fe2—C21	39.96 (11)	C18—C17—H17	125.7
C15—Fe2—C21	158.49 (11)	Fe2—C17—H17	125.7
C23—Fe2—C21	67.61 (12)	C19—C18—C17	108.0 (2)
C16—Fe2—C19	68.93 (9)	C19—C18—Fe2	69.48 (13)
C24—Fe2—C19	124.63 (11)	C17—C18—Fe2	69.50 (13)
C20—Fe2—C19	108.94 (10)	C19—C18—H18	126.0
C22—Fe2—C19	158.41 (11)	C17—C18—H18	126.0
C15—Fe2—C19	41.17 (9)	Fe2—C18—H18	126.0
C23—Fe2—C19	160.37 (11)	C18—C19—C15	108.3 (2)
C21—Fe2—C19	123.45 (11)	C18—C19—Fe2	70.20 (13)
C16—Fe2—C17	40.45 (9)	C15—C19—Fe2	69.26 (12)
C24—Fe2—C17	156.29 (12)	C18—C19—H19	125.8
C20—Fe2—C17	161.48 (12)	C15—C19—H19	125.8
C22—Fe2—C17	108.07 (10)	Fe2—C19—H19	125.8
C15—Fe2—C17	68.63 (9)	C21—C20—C24	107.7 (3)
C23—Fe2—C17	121.13 (11)	C21—C20—Fe2	70.00 (16)
C21—Fe2—C17	124.78 (11)	C24—C20—Fe2	69.61 (15)
C19—Fe2—C17	68.16 (9)	C21—C20—H20	126.2
C16—Fe2—C18	68.46 (9)	C24—C20—H20	126.2
C24—Fe2—C18	161.48 (12)	Fe2—C20—H20	126.2
C20—Fe2—C18	125.47 (12)	C22—C21—C20	107.9 (3)
C22—Fe2—C18	123.02 (10)	C22—C21—Fe2	69.87 (15)
C15—Fe2—C18	68.69 (9)	C20—C21—Fe2	69.68 (15)
C23—Fe2—C18	157.24 (11)	C22—C21—H21	126.1
C21—Fe2—C18	109.40 (11)	C20—C21—H21	126.1
C19—Fe2—C18	40.33 (9)	Fe2—C21—H21	126.1
C17—Fe2—C18	40.54 (9)	C21—C22—C23	108.9 (2)
C11—N1—N2	119.70 (19)	C21—C22—Fe2	70.17 (14)
C14—N2—N1	119.0 (2)	C23—C22—Fe2	70.07 (14)
C25—N3—N4	119.79 (19)	C21—C22—H22	125.6
C28—N4—N3	119.03 (18)	C23—C22—H22	125.6
C2—C1—C5	107.1 (2)	Fe2—C22—H22	125.6
C2—C1—C11	126.5 (2)	C22—C23—C24	107.6 (3)
C5—C1—C11	126.4 (2)	C22—C23—Fe2	69.84 (14)
C2—C1—Fe1	69.28 (13)	C24—C23—Fe2	69.57 (15)
C5—C1—Fe1	69.38 (13)	C22—C23—H23	126.2
C11—C1—Fe1	126.07 (16)	C24—C23—H23	126.2
C3—C2—C1	107.7 (2)	Fe2—C23—H23	126.2
C3—C2—Fe1	69.98 (14)	C23—C24—C20	108.0 (3)
C1—C2—Fe1	69.49 (13)	C23—C24—Fe2	70.07 (14)
C3—C2—H2	126.2	C20—C24—Fe2	69.86 (15)
C1—C2—H2	126.2	C23—C24—H24	126.0
Fe1—C2—H2	126.2	C20—C24—H24	126.0
C2—C3—C4	108.7 (2)	Fe2—C24—H24	126.0
C2—C3—Fe1	69.55 (14)	N3—C25—C26	121.5 (2)
C4—C3—Fe1	69.96 (14)	N3—C25—C15	115.79 (19)
C2—C3—H3	125.7	C26—C25—C15	122.74 (19)
C4—C3—H3	125.7	C27—C26—C25	118.6 (2)
Fe1—C3—H3	125.7	C27—C26—H26	120.7
C5—C4—C3	108.4 (2)	C25—C26—H26	120.7
C5—C4—Fe1	69.61 (14)	C26—C27—C28	116.4 (2)
C3—C4—Fe1	69.57 (14)	C26—C27—H27	121.8
C5—C4—H4	125.8	C28—C27—H27	121.8
C3—C4—H4	125.8	N4—C28—C27	124.7 (2)
Fe1—C4—H4	125.8	N4—C28—Cl2	115.15 (17)
C4—C5—C1	108.1 (2)	C27—C28—Cl2	120.14 (19)
			
C11—N1—N2—C14	−0.3 (3)	C12—C13—C14—Cl1	178.63 (18)
C25—N3—N4—C28	1.1 (3)	C24—Fe2—C15—C16	117.82 (16)
C7—Fe1—C1—C2	−116.75 (17)	C20—Fe2—C15—C16	159.42 (15)
C8—Fe1—C1—C2	−75.44 (18)	C22—Fe2—C15—C16	47.2 (3)
C6—Fe1—C1—C2	−158.78 (17)	C23—Fe2—C15—C16	76.58 (17)
C9—Fe1—C1—C2	−46.1 (4)	C21—Fe2—C15—C16	−170.2 (3)
C5—Fe1—C1—C2	118.6 (2)	C19—Fe2—C15—C16	−118.36 (19)
C3—Fe1—C1—C2	37.70 (14)	C17—Fe2—C15—C16	−37.53 (13)
C10—Fe1—C1—C2	171.0 (3)	C18—Fe2—C15—C16	−81.20 (14)
C4—Fe1—C1—C2	81.34 (15)	C16—Fe2—C15—C19	118.36 (19)
C7—Fe1—C1—C5	124.68 (18)	C24—Fe2—C15—C19	−123.82 (16)
C8—Fe1—C1—C5	165.98 (16)	C20—Fe2—C15—C19	−82.22 (18)
C2—Fe1—C1—C5	−118.6 (2)	C22—Fe2—C15—C19	165.5 (3)
C6—Fe1—C1—C5	82.64 (19)	C23—Fe2—C15—C19	−165.06 (14)
C9—Fe1—C1—C5	−164.7 (3)	C21—Fe2—C15—C19	−51.8 (3)
C3—Fe1—C1—C5	−80.88 (16)	C17—Fe2—C15—C19	80.83 (14)
C10—Fe1—C1—C5	52.4 (4)	C18—Fe2—C15—C19	37.16 (13)
C4—Fe1—C1—C5	−37.24 (15)	C16—Fe2—C15—C25	−121.1 (2)
C7—Fe1—C1—C11	4.0 (2)	C24—Fe2—C15—C25	−3.3 (2)
C8—Fe1—C1—C11	45.3 (2)	C20—Fe2—C15—C25	38.3 (2)
C2—Fe1—C1—C11	120.8 (2)	C22—Fe2—C15—C25	−74.0 (3)
C6—Fe1—C1—C11	−38.0 (2)	C23—Fe2—C15—C25	−44.6 (2)
C9—Fe1—C1—C11	74.7 (4)	C21—Fe2—C15—C25	68.7 (3)
C5—Fe1—C1—C11	−120.6 (3)	C19—Fe2—C15—C25	120.5 (2)
C3—Fe1—C1—C11	158.5 (2)	C17—Fe2—C15—C25	−158.7 (2)
C10—Fe1—C1—C11	−68.2 (4)	C18—Fe2—C15—C25	157.7 (2)
C4—Fe1—C1—C11	−157.9 (2)	C19—C15—C16—C17	0.6 (2)
C5—C1—C2—C3	−0.5 (3)	C25—C15—C16—C17	177.7 (2)
C11—C1—C2—C3	180.0 (2)	Fe2—C15—C16—C17	60.09 (16)
Fe1—C1—C2—C3	−59.82 (17)	C19—C15—C16—Fe2	−59.53 (15)
C5—C1—C2—Fe1	59.31 (15)	C25—C15—C16—Fe2	117.6 (2)
C11—C1—C2—Fe1	−120.2 (2)	C24—Fe2—C16—C17	160.71 (15)
C7—Fe1—C2—C3	−160.17 (16)	C20—Fe2—C16—C17	−169.5 (2)
C8—Fe1—C2—C3	−118.99 (17)	C22—Fe2—C16—C17	79.03 (17)
C6—Fe1—C2—C3	170.4 (3)	C15—Fe2—C16—C17	−119.01 (19)
C9—Fe1—C2—C3	−78.97 (18)	C23—Fe2—C16—C17	119.41 (15)
C5—Fe1—C2—C3	80.45 (16)	C21—Fe2—C16—C17	50.7 (3)
C1—Fe1—C2—C3	118.8 (2)	C19—Fe2—C16—C17	−80.64 (15)
C10—Fe1—C2—C3	−51.7 (4)	C18—Fe2—C16—C17	−37.22 (14)
C4—Fe1—C2—C3	37.20 (16)	C24—Fe2—C16—C15	−80.28 (16)
C7—Fe1—C2—C1	81.07 (17)	C20—Fe2—C16—C15	−50.5 (3)
C8—Fe1—C2—C1	122.25 (15)	C22—Fe2—C16—C15	−161.95 (13)
C6—Fe1—C2—C1	51.6 (3)	C23—Fe2—C16—C15	−121.58 (14)
C9—Fe1—C2—C1	162.27 (14)	C21—Fe2—C16—C15	169.7 (3)
C5—Fe1—C2—C1	−38.31 (14)	C19—Fe2—C16—C15	38.37 (13)
C3—Fe1—C2—C1	−118.8 (2)	C17—Fe2—C16—C15	119.01 (19)
C10—Fe1—C2—C1	−170.4 (3)	C18—Fe2—C16—C15	81.80 (14)
C4—Fe1—C2—C1	−81.56 (15)	C15—C16—C17—C18	−0.6 (3)
C1—C2—C3—C4	0.3 (3)	Fe2—C16—C17—C18	59.06 (16)
Fe1—C2—C3—C4	−59.20 (18)	C15—C16—C17—Fe2	−59.65 (15)
C1—C2—C3—Fe1	59.51 (16)	C24—Fe2—C17—C16	−44.9 (3)
C7—Fe1—C3—C2	44.9 (3)	C20—Fe2—C17—C16	167.2 (3)
C8—Fe1—C3—C2	77.75 (18)	C22—Fe2—C17—C16	−119.92 (15)
C6—Fe1—C3—C2	−167.9 (3)	C15—Fe2—C17—C16	38.28 (13)
C9—Fe1—C3—C2	119.82 (16)	C23—Fe2—C17—C16	−77.91 (17)
C5—Fe1—C3—C2	−82.93 (16)	C21—Fe2—C17—C16	−160.88 (15)
C1—Fe1—C3—C2	−38.39 (14)	C19—Fe2—C17—C16	82.71 (15)
C10—Fe1—C3—C2	160.64 (15)	C18—Fe2—C17—C16	120.1 (2)
C4—Fe1—C3—C2	−120.0 (2)	C16—Fe2—C17—C18	−120.1 (2)
C7—Fe1—C3—C4	164.9 (3)	C24—Fe2—C17—C18	−164.9 (2)
C8—Fe1—C3—C4	−162.27 (16)	C20—Fe2—C17—C18	47.2 (4)
C2—Fe1—C3—C4	120.0 (2)	C22—Fe2—C17—C18	120.03 (16)
C6—Fe1—C3—C4	−47.9 (4)	C15—Fe2—C17—C18	−81.78 (15)
C9—Fe1—C3—C4	−120.20 (17)	C23—Fe2—C17—C18	162.03 (15)
C5—Fe1—C3—C4	37.05 (15)	C21—Fe2—C17—C18	79.06 (18)
C1—Fe1—C3—C4	81.59 (16)	C19—Fe2—C17—C18	−37.34 (14)
C10—Fe1—C3—C4	−79.38 (19)	C16—C17—C18—C19	0.4 (3)
C2—C3—C4—C5	0.0 (3)	Fe2—C17—C18—C19	58.98 (16)
Fe1—C3—C4—C5	−58.93 (17)	C16—C17—C18—Fe2	−58.58 (16)
C2—C3—C4—Fe1	58.95 (18)	C16—Fe2—C18—C19	−82.41 (15)
C7—Fe1—C4—C5	−39.2 (4)	C24—Fe2—C18—C19	41.3 (4)
C8—Fe1—C4—C5	161.4 (2)	C20—Fe2—C18—C19	77.08 (18)
C2—Fe1—C4—C5	82.70 (16)	C22—Fe2—C18—C19	161.46 (15)
C6—Fe1—C4—C5	−76.7 (2)	C15—Fe2—C18—C19	−37.91 (13)
C9—Fe1—C4—C5	−161.44 (15)	C23—Fe2—C18—C19	−162.6 (3)
C1—Fe1—C4—C5	38.20 (14)	C21—Fe2—C18—C19	119.22 (15)
C3—Fe1—C4—C5	119.9 (2)	C17—Fe2—C18—C19	−119.5 (2)
C10—Fe1—C4—C5	−118.86 (16)	C16—Fe2—C18—C17	37.13 (14)
C7—Fe1—C4—C3	−159.2 (4)	C24—Fe2—C18—C17	160.8 (3)
C8—Fe1—C4—C3	41.5 (3)	C20—Fe2—C18—C17	−163.38 (16)
C2—Fe1—C4—C3	−37.21 (16)	C22—Fe2—C18—C17	−79.00 (18)
C6—Fe1—C4—C3	163.43 (18)	C15—Fe2—C18—C17	81.63 (15)
C9—Fe1—C4—C3	78.64 (19)	C23—Fe2—C18—C17	−43.0 (3)
C5—Fe1—C4—C3	−119.9 (2)	C21—Fe2—C18—C17	−121.24 (16)
C1—Fe1—C4—C3	−81.71 (16)	C19—Fe2—C18—C17	119.5 (2)
C10—Fe1—C4—C3	121.23 (17)	C17—C18—C19—C15	0.0 (3)
C3—C4—C5—C1	−0.3 (3)	Fe2—C18—C19—C15	58.95 (15)
Fe1—C4—C5—C1	−59.24 (16)	C17—C18—C19—Fe2	−58.99 (16)
C3—C4—C5—Fe1	58.90 (18)	C16—C15—C19—C18	−0.3 (2)
C2—C1—C5—C4	0.5 (3)	C25—C15—C19—C18	−177.5 (2)
C11—C1—C5—C4	−180.0 (2)	Fe2—C15—C19—C18	−59.52 (16)
Fe1—C1—C5—C4	59.77 (17)	C16—C15—C19—Fe2	59.20 (15)
C2—C1—C5—Fe1	−59.25 (16)	C25—C15—C19—Fe2	−118.0 (2)
C11—C1—C5—Fe1	120.3 (2)	C16—Fe2—C19—C18	81.13 (15)
C7—Fe1—C5—C4	166.37 (17)	C24—Fe2—C19—C18	−165.25 (16)
C8—Fe1—C5—C4	−157.4 (3)	C20—Fe2—C19—C18	−122.94 (16)
C2—Fe1—C5—C4	−80.92 (16)	C22—Fe2—C19—C18	−46.5 (3)
C6—Fe1—C5—C4	123.53 (18)	C15—Fe2—C19—C18	119.60 (19)
C9—Fe1—C5—C4	46.1 (4)	C23—Fe2—C19—C18	159.8 (3)
C1—Fe1—C5—C4	−119.2 (2)	C21—Fe2—C19—C18	−80.62 (18)
C3—Fe1—C5—C4	−37.31 (15)	C17—Fe2—C19—C18	37.53 (14)
C10—Fe1—C5—C4	81.17 (19)	C16—Fe2—C19—C15	−38.47 (13)
C7—Fe1—C5—C1	−74.4 (2)	C24—Fe2—C19—C15	75.15 (18)
C8—Fe1—C5—C1	−38.2 (4)	C20—Fe2—C19—C15	117.46 (16)
C2—Fe1—C5—C1	38.31 (14)	C22—Fe2—C19—C15	−166.1 (3)
C6—Fe1—C5—C1	−117.24 (18)	C23—Fe2—C19—C15	40.2 (4)
C9—Fe1—C5—C1	165.3 (3)	C21—Fe2—C19—C15	159.78 (15)
C3—Fe1—C5—C1	81.92 (15)	C17—Fe2—C19—C15	−82.07 (14)
C10—Fe1—C5—C1	−159.60 (16)	C18—Fe2—C19—C15	−119.60 (19)
C4—Fe1—C5—C1	119.2 (2)	C16—Fe2—C20—C21	−159.7 (2)
C7—Fe1—C6—C10	118.5 (2)	C24—Fe2—C20—C21	−118.7 (2)
C8—Fe1—C6—C10	80.81 (18)	C22—Fe2—C20—C21	−37.46 (17)
C2—Fe1—C6—C10	159.0 (2)	C15—Fe2—C20—C21	163.35 (16)
C9—Fe1—C6—C10	37.38 (17)	C23—Fe2—C20—C21	−80.95 (18)
C5—Fe1—C6—C10	−119.59 (18)	C19—Fe2—C20—C21	119.75 (17)
C1—Fe1—C6—C10	−163.40 (16)	C17—Fe2—C20—C21	42.1 (4)
C3—Fe1—C6—C10	−41.4 (4)	C18—Fe2—C20—C21	77.9 (2)
C4—Fe1—C6—C10	−77.9 (2)	C16—Fe2—C20—C24	−41.0 (3)
C8—Fe1—C6—C7	−37.65 (17)	C22—Fe2—C20—C24	81.23 (18)
C2—Fe1—C6—C7	40.5 (3)	C15—Fe2—C20—C24	−77.96 (18)
C9—Fe1—C6—C7	−81.08 (18)	C23—Fe2—C20—C24	37.74 (16)
C5—Fe1—C6—C7	121.94 (17)	C21—Fe2—C20—C24	118.7 (2)
C1—Fe1—C6—C7	78.14 (19)	C19—Fe2—C20—C24	−121.55 (16)
C3—Fe1—C6—C7	−159.9 (3)	C17—Fe2—C20—C24	160.8 (3)
C10—Fe1—C6—C7	−118.5 (2)	C18—Fe2—C20—C24	−163.38 (16)
C4—Fe1—C6—C7	163.59 (17)	C24—C20—C21—C22	0.0 (3)
C10—C6—C7—C8	−0.1 (3)	Fe2—C20—C21—C22	59.66 (18)
Fe1—C6—C7—C8	59.90 (19)	C24—C20—C21—Fe2	−59.65 (18)
C10—C6—C7—Fe1	−60.03 (19)	C16—Fe2—C21—C22	38.4 (4)
C2—Fe1—C7—C8	78.14 (19)	C24—Fe2—C21—C22	−80.97 (18)
C6—Fe1—C7—C8	−118.9 (2)	C20—Fe2—C21—C22	−119.0 (2)
C9—Fe1—C7—C8	−37.75 (17)	C15—Fe2—C21—C22	−160.4 (2)
C5—Fe1—C7—C8	162.51 (16)	C23—Fe2—C21—C22	−37.19 (16)
C1—Fe1—C7—C8	120.98 (17)	C19—Fe2—C21—C22	161.23 (15)
C3—Fe1—C7—C8	46.2 (3)	C17—Fe2—C21—C22	76.05 (19)
C10—Fe1—C7—C8	−81.15 (18)	C18—Fe2—C21—C22	118.63 (17)
C4—Fe1—C7—C8	−167.6 (3)	C16—Fe2—C21—C20	157.4 (3)
C8—Fe1—C7—C6	118.9 (2)	C24—Fe2—C21—C20	38.00 (17)
C2—Fe1—C7—C6	−162.93 (17)	C22—Fe2—C21—C20	119.0 (2)
C9—Fe1—C7—C6	81.18 (19)	C15—Fe2—C21—C20	−41.4 (4)
C5—Fe1—C7—C6	−78.6 (2)	C23—Fe2—C21—C20	81.78 (19)
C1—Fe1—C7—C6	−120.09 (17)	C19—Fe2—C21—C20	−79.8 (2)
C3—Fe1—C7—C6	165.1 (3)	C17—Fe2—C21—C20	−164.97 (16)
C10—Fe1—C7—C6	37.78 (17)	C18—Fe2—C21—C20	−122.40 (18)
C4—Fe1—C7—C6	−48.6 (4)	C20—C21—C22—C23	0.0 (3)
C6—C7—C8—C9	0.2 (3)	Fe2—C21—C22—C23	59.58 (18)
Fe1—C7—C8—C9	60.09 (19)	C20—C21—C22—Fe2	−59.54 (18)
C6—C7—C8—Fe1	−59.86 (19)	C16—Fe2—C22—C21	−165.08 (16)
C7—Fe1—C8—C9	−118.6 (3)	C24—Fe2—C22—C21	81.84 (19)
C2—Fe1—C8—C9	122.90 (18)	C20—Fe2—C22—C21	37.80 (18)
C6—Fe1—C8—C9	−80.5 (2)	C15—Fe2—C22—C21	159.7 (3)
C5—Fe1—C8—C9	−166.7 (3)	C23—Fe2—C22—C21	119.8 (2)
C1—Fe1—C8—C9	164.34 (16)	C19—Fe2—C22—C21	−46.9 (3)
C3—Fe1—C8—C9	81.8 (2)	C17—Fe2—C22—C21	−123.02 (17)
C10—Fe1—C8—C9	−36.95 (18)	C18—Fe2—C22—C21	−80.87 (19)
C4—Fe1—C8—C9	51.9 (3)	C16—Fe2—C22—C23	75.13 (19)
C2—Fe1—C8—C7	−118.51 (19)	C24—Fe2—C22—C23	−37.94 (17)
C6—Fe1—C8—C7	38.10 (19)	C20—Fe2—C22—C23	−81.99 (19)
C9—Fe1—C8—C7	118.6 (3)	C15—Fe2—C22—C23	39.9 (4)
C5—Fe1—C8—C7	−48.1 (4)	C21—Fe2—C22—C23	−119.8 (2)
C1—Fe1—C8—C7	−77.1 (2)	C19—Fe2—C22—C23	−166.6 (2)
C3—Fe1—C8—C7	−159.65 (18)	C17—Fe2—C22—C23	117.19 (17)
C10—Fe1—C8—C7	81.6 (2)	C18—Fe2—C22—C23	159.35 (16)
C4—Fe1—C8—C7	170.5 (3)	C21—C22—C23—C24	−0.1 (3)
C7—C8—C9—C10	−0.2 (3)	Fe2—C22—C23—C24	59.57 (18)
Fe1—C8—C9—C10	59.47 (19)	C21—C22—C23—Fe2	−59.65 (18)
C7—C8—C9—Fe1	−59.70 (18)	C16—Fe2—C23—C22	−122.40 (17)
C7—Fe1—C9—C8	38.04 (18)	C24—Fe2—C23—C22	118.7 (2)
C2—Fe1—C9—C8	−74.2 (2)	C20—Fe2—C23—C22	80.79 (19)
C6—Fe1—C9—C8	82.5 (2)	C15—Fe2—C23—C22	−164.31 (15)
C5—Fe1—C9—C8	168.2 (3)	C21—Fe2—C23—C22	37.07 (17)
C1—Fe1—C9—C8	−39.8 (4)	C19—Fe2—C23—C22	165.3 (3)
C3—Fe1—C9—C8	−116.20 (18)	C17—Fe2—C23—C22	−81.09 (19)
C10—Fe1—C9—C8	120.1 (3)	C18—Fe2—C23—C22	−49.9 (3)
C4—Fe1—C9—C8	−158.16 (17)	C16—Fe2—C23—C24	118.90 (18)
C7—Fe1—C9—C10	−82.0 (2)	C20—Fe2—C23—C24	−37.90 (17)
C8—Fe1—C9—C10	−120.1 (3)	C22—Fe2—C23—C24	−118.7 (2)
C2—Fe1—C9—C10	165.73 (18)	C15—Fe2—C23—C24	77.0 (2)
C6—Fe1—C9—C10	−37.6 (2)	C21—Fe2—C23—C24	−81.62 (19)
C5—Fe1—C9—C10	48.1 (4)	C19—Fe2—C23—C24	46.6 (4)
C1—Fe1—C9—C10	−159.9 (3)	C17—Fe2—C23—C24	160.22 (17)
C3—Fe1—C9—C10	123.72 (19)	C18—Fe2—C23—C24	−168.6 (2)
C4—Fe1—C9—C10	81.8 (2)	C22—C23—C24—C20	0.1 (3)
C8—C9—C10—C6	0.2 (3)	Fe2—C23—C24—C20	59.82 (18)
Fe1—C9—C10—C6	59.28 (19)	C22—C23—C24—Fe2	−59.74 (18)
C8—C9—C10—Fe1	−59.13 (19)	C21—C20—C24—C23	−0.1 (3)
C7—C6—C10—C9	0.0 (3)	Fe2—C20—C24—C23	−59.96 (18)
Fe1—C6—C10—C9	−59.4 (2)	C21—C20—C24—Fe2	59.90 (18)
C7—C6—C10—Fe1	59.36 (18)	C16—Fe2—C24—C23	−78.29 (19)
C7—Fe1—C10—C9	80.8 (2)	C20—Fe2—C24—C23	118.9 (2)
C8—Fe1—C10—C9	37.03 (18)	C22—Fe2—C24—C23	37.69 (17)
C2—Fe1—C10—C9	−36.8 (4)	C15—Fe2—C24—C23	−121.07 (17)
C6—Fe1—C10—C9	119.2 (3)	C21—Fe2—C24—C23	81.05 (18)
C5—Fe1—C10—C9	−160.68 (17)	C19—Fe2—C24—C23	−162.73 (16)
C1—Fe1—C10—C9	160.5 (3)	C17—Fe2—C24—C23	−46.1 (3)
C3—Fe1—C10—C9	−75.3 (2)	C18—Fe2—C24—C23	166.0 (3)
C4—Fe1—C10—C9	−118.02 (18)	C16—Fe2—C24—C20	162.85 (15)
C7—Fe1—C10—C6	−38.41 (18)	C22—Fe2—C24—C20	−81.16 (18)
C8—Fe1—C10—C6	−82.2 (2)	C15—Fe2—C24—C20	120.07 (16)
C2—Fe1—C10—C6	−156.1 (3)	C23—Fe2—C24—C20	−118.9 (2)
C9—Fe1—C10—C6	−119.2 (3)	C21—Fe2—C24—C20	−37.81 (16)
C5—Fe1—C10—C6	80.1 (2)	C19—Fe2—C24—C20	78.41 (19)
C1—Fe1—C10—C6	41.3 (4)	C17—Fe2—C24—C20	−164.9 (2)
C3—Fe1—C10—C6	165.43 (18)	C18—Fe2—C24—C20	47.2 (4)
C4—Fe1—C10—C6	122.73 (19)	N4—N3—C25—C26	−1.1 (3)
N2—N1—C11—C12	−0.9 (3)	N4—N3—C25—C15	178.98 (19)
N2—N1—C11—C1	178.51 (19)	C16—C15—C25—N3	170.2 (2)
C2—C1—C11—N1	−170.4 (2)	C19—C15—C25—N3	−13.2 (3)
C5—C1—C11—N1	10.1 (3)	Fe2—C15—C25—N3	−101.6 (2)
Fe1—C1—C11—N1	99.8 (2)	C16—C15—C25—C26	−9.7 (3)
C2—C1—C11—C12	8.9 (4)	C19—C15—C25—C26	166.9 (2)
C5—C1—C11—C12	−170.5 (2)	Fe2—C15—C25—C26	78.5 (3)
Fe1—C1—C11—C12	−80.8 (3)	N3—C25—C26—C27	0.2 (3)
N1—C11—C12—C13	1.1 (3)	C15—C25—C26—C27	−179.9 (2)
C1—C11—C12—C13	−178.2 (2)	C25—C26—C27—C28	0.6 (3)
C11—C12—C13—C14	−0.3 (3)	N3—N4—C28—C27	−0.3 (4)
N1—N2—C14—C13	1.2 (4)	N3—N4—C28—Cl2	−179.20 (16)
N1—N2—C14—Cl1	−178.37 (17)	C26—C27—C28—N4	−0.6 (4)
C12—C13—C14—N2	−0.9 (4)	C26—C27—C28—Cl2	178.28 (18)

### Hydrogen-bond geometry (Å, º)

**Table:**

*D*—H···*A*	*D*—H	H···*A*	*D*···*A*	*D*—H···*A*
C2—H2···N3^i^	0.98	2.55	3.454 (4)	153
C12—H12···N4^i^	0.93	2.41	3.320 (4)	166
C26—H26···N2^ii^	0.93	2.43	3.302 (4)	156

Symmetry codes: (i) −*x*+1, −*y*+1, −*z*+1; (ii) −*x*+1/2, −*y*+1/2, −*z*+1.

## References

[bb1] Agilent (2011). *CrysAlis PRO.* Agilent Technologies, Abingdon, England.

[bb2] Beletskaya, I. P., Tsvetkov, A. V., Latyshev, G. V., Tafeenko, V. A. & Lukashev, N. V. (2001). *J. Organomet. Chem.* **637–639**, 653–663.

[bb3] Meijere, A. D. & Diederich, F. (2004). *Metal-catalyzed Cross-coupling Reactions.* 2nd ed. Weinheim: Wiley-VCH.

[bb4] Sheldrick, G. M. (2008). *Acta Cryst.* A**64**, 112–122. 10.1107/S0108767307043930 18156677

[bb5] Tsvetkov, A. V., Tsvetkov, A. V., Lukashev, N. V. & Beletskaya, I. P. (2000). *Tetrahedron Lett.* **41**, 3987–3990.

[bb6] Xu, C., Li, H. M., Wang, Z. Q., Fu, W. J., Zhang, Y. Q. & Ji, B. M. (2012). *Aust. J. Chem.* **65**, 366–370.

[bb7] Xu, C., Zhang, Y. P., Wang, Z. Q., Fu, W. J., Hao, X. Q., Xu, Y. & Ji, B. M. (2010). *Chem. Commun.* **46**, 6852–6854. 10.1039/c0cc01870h 20730199

